# Brain structure analysis of different age groups of Diannan small‐ear pigs

**DOI:** 10.1002/ibra.12058

**Published:** 2022-08-13

**Authors:** Yi‐Fan Liu, Chang‐Le Fang, Yu Pi, Teng‐Fei Ke, Ji‐Xiang Chu, Lin‐Na Tang, Lan‐Chun Zhang, Somjit Wanchana, Cheng‐De Liao

**Affiliations:** ^1^ Department of Radiology, The Third Affiliated Hospital of Kunming Medical University Yunnan Cancer Hospital Kunming China; ^2^ School of Anesthesiology Southwest Medical University Luzhou China; ^3^ Department of Laboratory Animal, Institute of Neuroscience Kunming Medical University Kunming China; ^4^ Boromarajonani College of Nursing Ratchaburi Thailand

**Keywords:** brain structure, Diannan small‐ear pig, magnetic resonance imaging

## Abstract

The objective of the study is to investigate the brain development and atrophy of Diannan small‐ear pigs in different ages using magnetic resonance imaging (MRI). A total of 12 Diannan small‐ear pigs were included and divided into the young group, adult group, and middle‐and‐old age (M&O) group according to their age. The brain structure of pigs was scanned using MRI, and the brain data obtained were statistically analyzed by signal conversion and image reconstruction. Compared with the young group, the signals of most brain structures in the adult group and M&O group were significantly decreased (*p* < 0.05). Compared with the adult group, the signal intensity of the right caudate nucleus and the right lateral ventricle in the M&O group was significantly increased, while the signal intensity of other regions was almost significantly decreased (*p* < 0.05). Compared with the young group, both adult and M&O groups had some degree of brain atrophy. Brain atrophy in the precuneus and the inferior temporal gyrus was more predominant in the M&O group in comparison with the adult group. The present study demonstrated that the brain signal of Diannan small‐ear pigs gradually diminished with age, while the degree of brain atrophy was the opposite, providing the basic data on the brain of Diannan small‐ear pigs.

## Graphical abstract

In this study, structural brain images of three groups of Diannnan small‐ear pigs of different ages were scanned by magnetic resonance imaging, 3D images were constructed for whole‐brain images and VBM analysis of T2 structural images was performed, and the results were compared cross‐sectionally and longitudinally. The results revealed that brain development and brain atrophy were closely related to age. In this study, the basic brain tissue data were measured in Diannnan small‐ear pigs to provide basic data for the development of brain test standards, related studies, and modeling of related brain diseases in Diannnan small‐ear pigs.

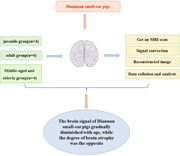

## 1 INTRODUCTION

In recent years, researchers have become increasingly aware of the limitations of some experimental animal models. It is, therefore, of great significance to select experimental animals with similar features of function, metabolism, structure, and disease, and at an appropriate cost. Generally, the higher the degree of evolution of laboratory animals, the more complex their metabolism, function, and structure are, and the closer the models and drug experiments are more similar to human beings.^1^ At present, mice are still the most widely used model animal, but there are great differences between mice and humans, and there are great limitations in the study of some specific diseases. Nonhuman primates, such as monkeys and gorillas, are the ideal model animals that are closest to human beings.^2^, ^3^ However, these animals are not widely used in clinical practice because of their high cost and ethical issues. Due to the advantages of minimal differences from humans, manageable costs, and no major ethical issues,^4^ some researchers have suggested that miniature pigs are the most valuable experimental model animals, except for nonhuman primates.^5^ Laboratory miniature pigs are now widely used in biomedical research, as miniature pigs are similar to humans in terms of anatomy, physiology, biochemistry, and disease occurrence and development, scientific research investment is increasing, and the number of miniature pigs used for experimental research is also on the increase.^6^, ^7^ China has abundant resources of miniature pigs, and different strains of miniature pigs have unique biological characteristics. Moreover, it is extremely insufficient to investigate the Diannan small‐ear pigs in comparison with Guizhou miniature pigs, Bama miniature pigs, Minnesota miniature pigs, and Bittman−Moor miniature pigs.^8^, ^9^ Therefore, it is important to supplement the biological characteristics of Diannan small‐ear pigs, explore their experimental research value, and construct corresponding disease animal models for biomedical studies.^10^, ^11^

In the present study, we conducted brain structure scans on 12 Diannan small‐ear pigs of different ages and determined the basic data of the brain tissues using magnetic resonance imaging (MRI), providing the basic data on the brain development of Diannan small‐ear pigs and the establishment of the brain‐related diseases.

## 2 MATERIALS AND METHODS

### 2.1 Animals and grouping

The Diannan small‐ear pigs (*n* = 12) were provided by the Department of Laboratory Animal of Kunming Medical University. The previous studies show that the young pigs are aged 2−12 months, adult pigs are aged over 12 months, and the old pigs are aged over 72 months. Therefore, in this study, the pigs were divided into the young group (*n* = 4, aged 2−3 months), adult group (*n* = 4, aged 16−19 months), and middle‐and‐old age (M&O) group (*n* = 4, aged 53−68 months) (Table 1) according to the different age. The study was approved by the Animal Committee of Kunming Medical University (no. kmmu2021570), and all procedures followed the regulations of the China Animal Protection and Ethics Committee for Laboratory Animals.

**Table 1:** Animal grouping

Items	Young group (*n* = 4)		Adult group (*n* = 4)		M&O group (*n* = 4)	
	Mean ± SD	Range	Mean ± SD	Range	Mean ± SD	Range
Age, months	2.5 ± 0.58	2−3	17.5 ± 1.73	16−19	60 ± 8.12	53−68
Weight, kg	3.38 ± 0.32	3.0−3.75	71.13 ± 11.23	61.3−81.2	88.55 ± 4.26	82.3−91.8

Abbreviation: M&O, middle‐and‐old age pigs.

### 2.2 MRI of the brains of Diannan small‐ear pigs

#### 2.2.1 Animals and pre‐MRI preparation

All MRI scans were performed using a 3.0T MRI scanner (Ingenia 3.0; Philips) to collect MRI images of the brains of Diannan small‐ear pigs. The pigs were anesthetized with 3% pentobarbital sodium and were then placed on the scanning bed. A horizontal scanning frame with an inner diameter of 16cm and a volume or surface coil for scanning the Diannan small‐ear pigs were used in the process. The physiological parameters of the experimental animals were continuously and dynamically monitored, including the heart rate (HR), respiratory rate (RR), temperature (T), and so forth. Body temperature was maintained at about 37°C with a heating blanket.

#### 2.2.2 MRI examination

The MRI scan parameters of the brain structures of Diannan small‐ear pigs were set as follows: (1) coronal T2‐weighted images (T2WI): rapid imaging with refocused echo (RIRE); (2) time repetition (TR): 5750 ms; (3) echo time (TE): 12 ms; (4) field of view (FOV): 3.2 cm × 3.2 cm, layer thickness 0.5 mm, layer spacing 0 mm, 50 layers, matrix size 256 × 256, spatial resolution 0.125 × 0.125, average number of times is 2; (5) scanning time: 6 min.

Digital imaging and communications in medicine (DICOM) images were converted to NIFI format using the Micron software package, and all voxels were expanded 10 times for subsequent processing by SPM12 using Data Processing & Analysis for Brain Imaging (DPABI) software for all structural image data (http://www.fil.ion.ucl.ac.uk/spm/software/spm12/). Using the display function of SPM12, the structural images were converted to the same spatial orientation as the structural image template of the pigs.^2^ Whole‐brain images were delineated using ITK‐SNAP (http://www.itksnap.org/pmwiki/pmwiki.php), and regions of interest (ROI) were delineated and their volumes and signal values were measured based on stereograms and histological maps of the pigs.^3^ The NIFI whole‐brain images were converted to MZ3 format using the Surf‐Ice package (https://www.nitrc.org/projects/surfice/), and the BrainNet Viewer (https://www.nitrc.org/projects/bnv/) path in MATLAB was used for 3D reconstruction. SPM12 software was used to perform VBM analysis based on T2 structural images, as well as to calculate the volume of gray matter and white matter cerebrospinal fluid (CSF) as follows: T2 images were converted to standard space, spatial segmentation of T2 images was performed to segment white matter, gray matter, and CSF, templates were made using rigid‐body transformed RC1/RC2/RC3, and the template files were converted to standard space template of the pigs for statistical analysis.

### 2.3 Statistical analysis

All data were entered into the SPSS 21.0 statistical software for statistical analysis. One‐way ANOVA was used for the quantitative data, and the results were expressed as mean ± standard deviation. *p < 0.05 indicated statistically significant difference. The statistical analysis module of SPM12 software was used for the statistical analysis of VBM data. Two samples *t* test was used for comparison between the two groups, and Gaussian random field theory was used to correct for multiple comparisons. The test level of each voxel was set as *p* < 0.001. The one‐tailed *t* test was used, and the level of the mass test was set as *p* < 0.05. The results were presented using DPABI Viewer.

## 3 RESULTS

### 3.1 Comparison of T2WI signal intensity in brain regions of Diannan small‐ear pigs

**Figure 1:**
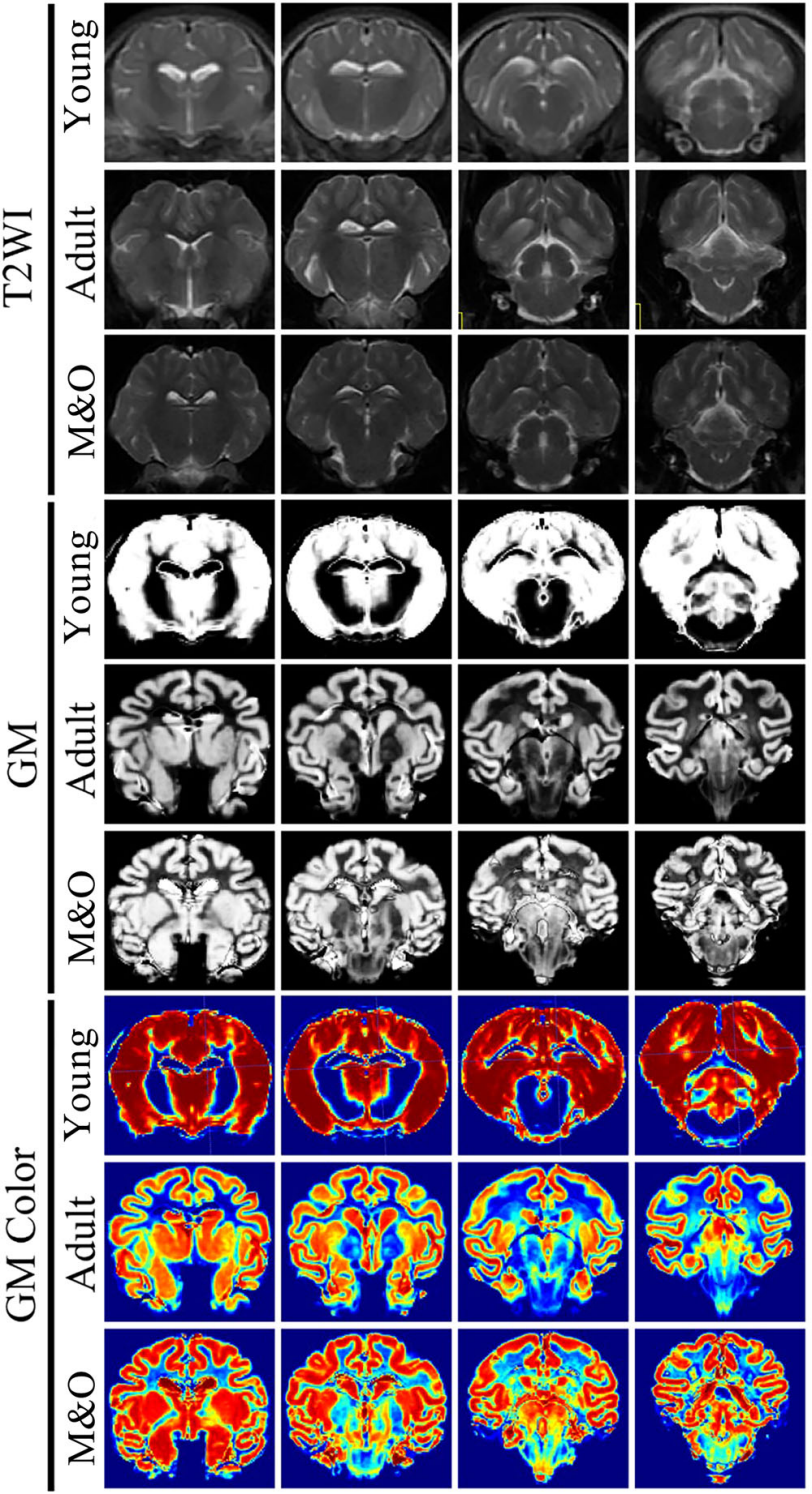
Comparison of T2WI signal intensity in brain regions of Diannan small‐ear pigs. From top to bottom, the T2WI, gray matter segmentation original image, and gray matter segmentation color image in the young group, adult group, and M&O group are shown. GM, gray matter; GM color, gray matter image; M&O, middle‐and‐old age pigs; T2WI, T2‐weighted imaging. [Color figure can be viewed at wileyonlinelibrary.com]

**Figure 2:**
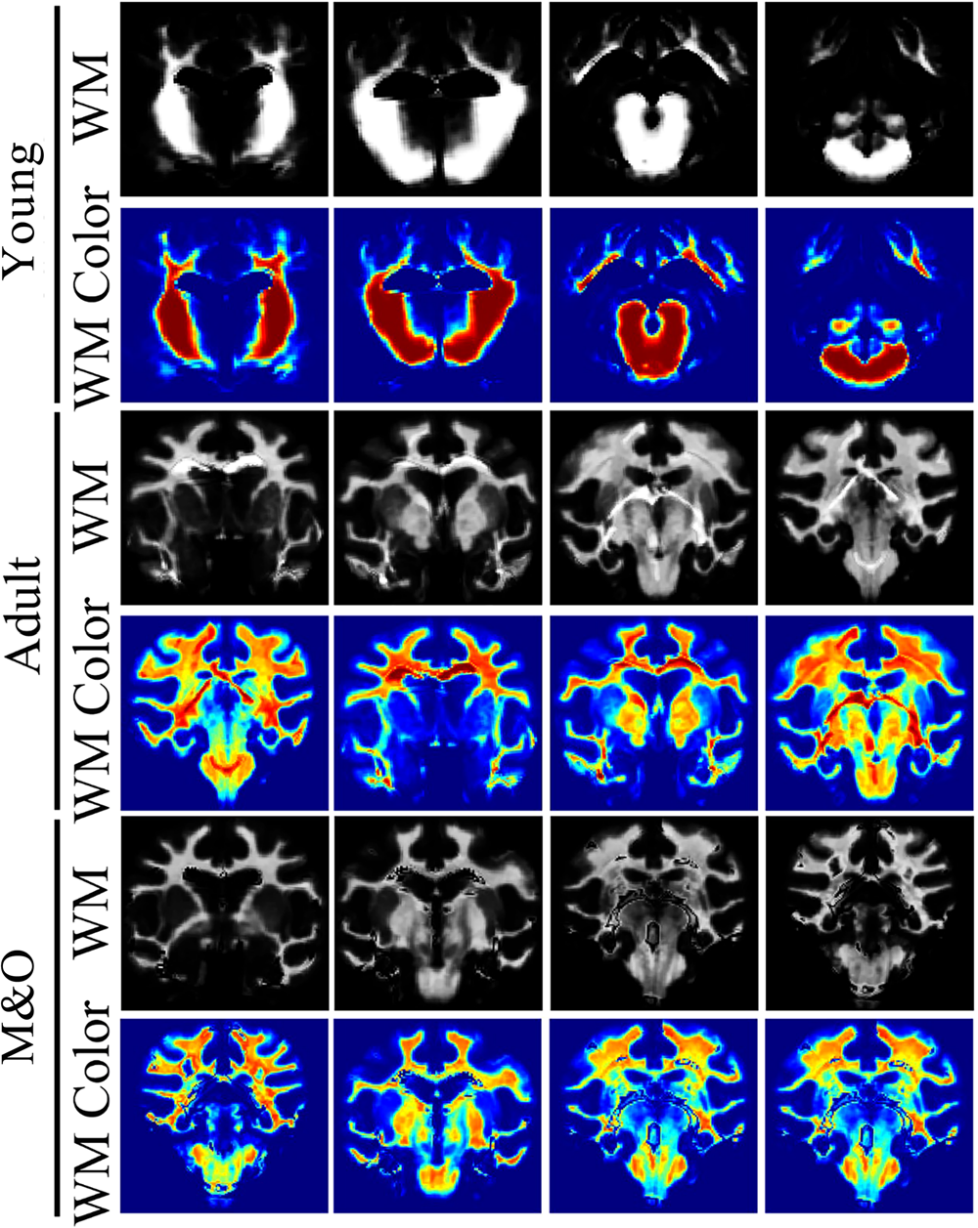
White matter image of segmented brain tissue in T2WI of Diannan small‐ear pigs. From top to bottom, the young group, adult group, and M&O group were successively divided into the original WM segmentation image and the color WM segmentation image. M&O, middle‐and‐old age pigs; T2WI, T2‐weighted imaging; WM, white matter. [Color figure can be viewed at wileyonlinelibrary.com]

**Figure 3:**
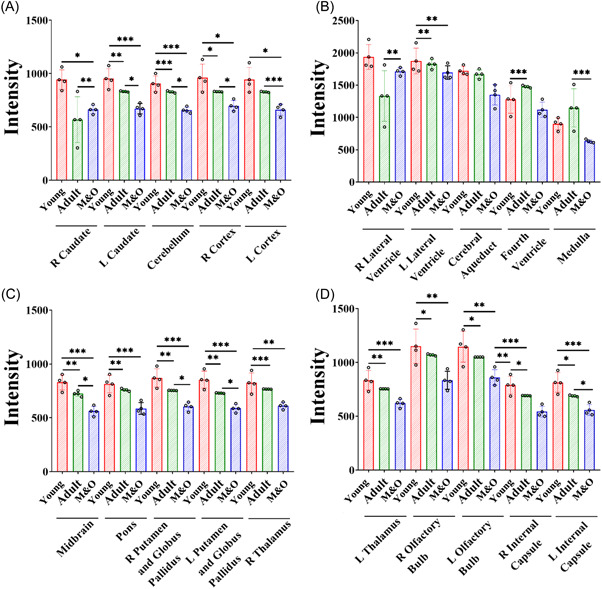
Comparison of signal intensity of brain regions in T2WI of Diannan small‐ear pigs. (A) Comparison of the signal intensity of left and right caudate nucleus, cerebellum, and left and right cortices between the young group, the adult group, and the M&O group. (B) Comparison of signal intensity between the left and right lateral ventricles, midbrain aqueduct, fourth ventricle, and medulla oblongata in the young group, the adult group, and the M&O group. (C) Comparison of signal intensity of left and right putamen pallidus, midbrain, and right thalamus between the young group, the adult group, and the M&O group. (D) Comparison of signal intensity of left thalamus, left and right olfactory bulb, and internal capsule between the young group, the adult group, and the M&O group. M&O, middle‐and‐old age pigs; T2WI, T2‐weighted imaging.

Compared with the young group, the signal intensity of the left and right caudate nucleus, cerebellum, left and right cortex, left lateral ventricle, midbrain, pons, right and left pallidus putamen, right and left thalamus, left and right olfactory bulb, left and right internal capsule in the adult group and M&O group were significantly decreased (*p* < 0.05, Figures 1, 2, 3). Compared with the adult group, the signal intensity of the right caudate nucleus and right lateral ventricle in the M&O group was significantly increased; however, the signal intensity of the left caudate nucleus, cerebellum, left and right cortical cortex, medulla oblongata, midbrain, left and right putamen pallidus, and left and right internal capsule were significantly decreased (*p* < 0.05, Figures 1, 2, 3). In addition, the signal intensity of the left and the right internal capsule was significantly reduced in the M&O group in comparison with the adult group.

### 3.2 Morphological study of Diannan small‐ear pig based on voxel analysis

**Figure 4:**
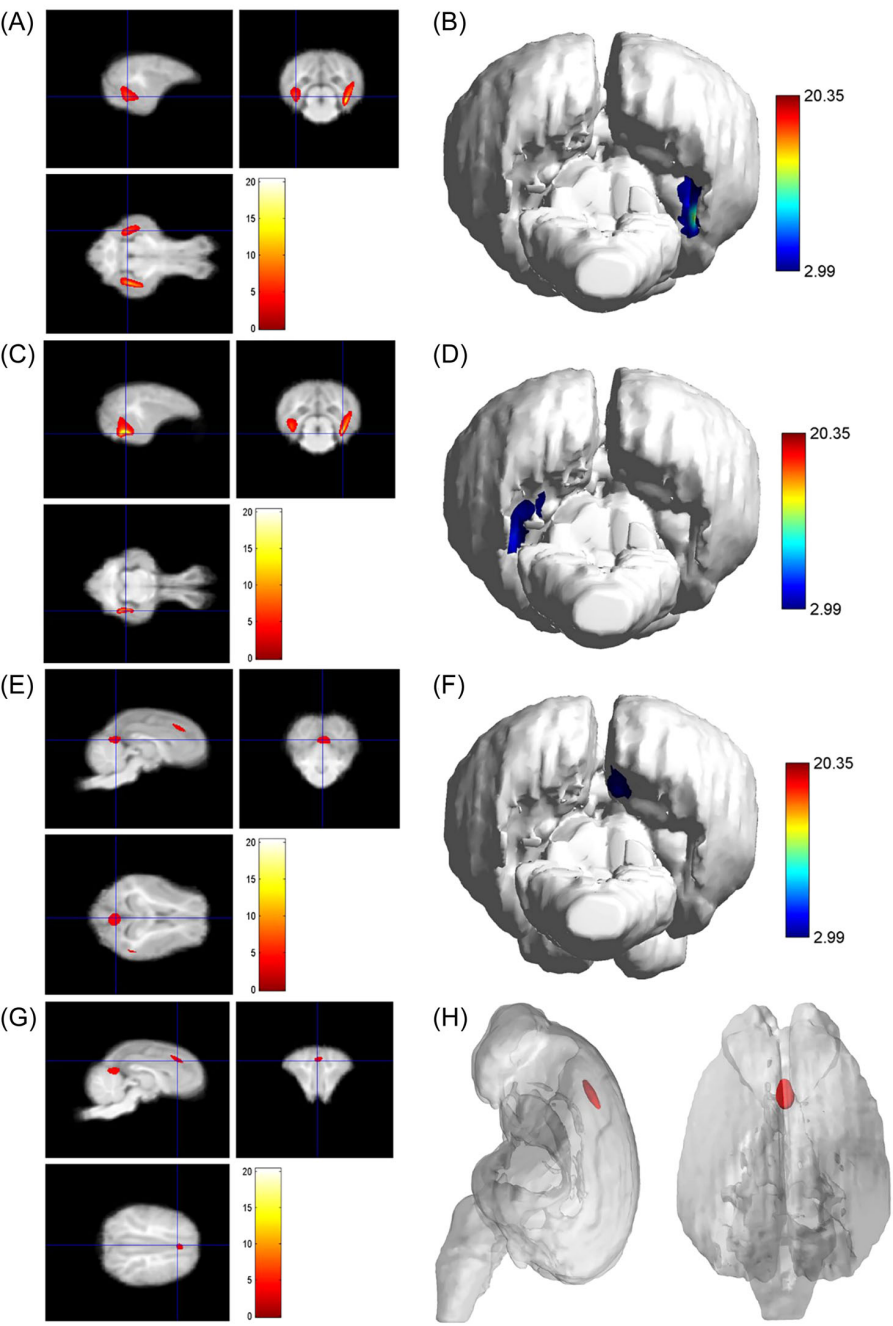
Results of the volume of young and adult groups. (A) In the adult group, the atrophy in the right occipital lobe, the main peak area (glossal gyrus; *x* = 16.5, *y* = −3, *z* = −0.5), and the total cluster size was 3953. (B) The 3D rendering of the atrophy area of the right occipital lobe. (C) Atrophy of the left occipital lobe in the main peak region (glossal gyrus; *x* = −14, *y* = −7, *z* = −2.5), and the total cluster size was 2281. (D) The 3D rendering of the atrophy area of the left occipital lobe. (E) Atrophy of the right precuneus primary peak region (precuneus; *x* = 0.5, *y* = −13, *z* = 5.5), and the total cluster size was 814. (F) The 3D rendering of the right precuneus atrophy area. (G) Atrophy in the right superior frontal gyrus main peak region (superior frontal gyrus; *x* = 0.5, *y* = 21, *z* = 12.5), and the total cluster size was 333. (H) The 3D reconstruction of the atrophy area of the right superior frontal gyrus. [Color figure can be viewed at wileyonlinelibrary.com]

**Figure 5:**
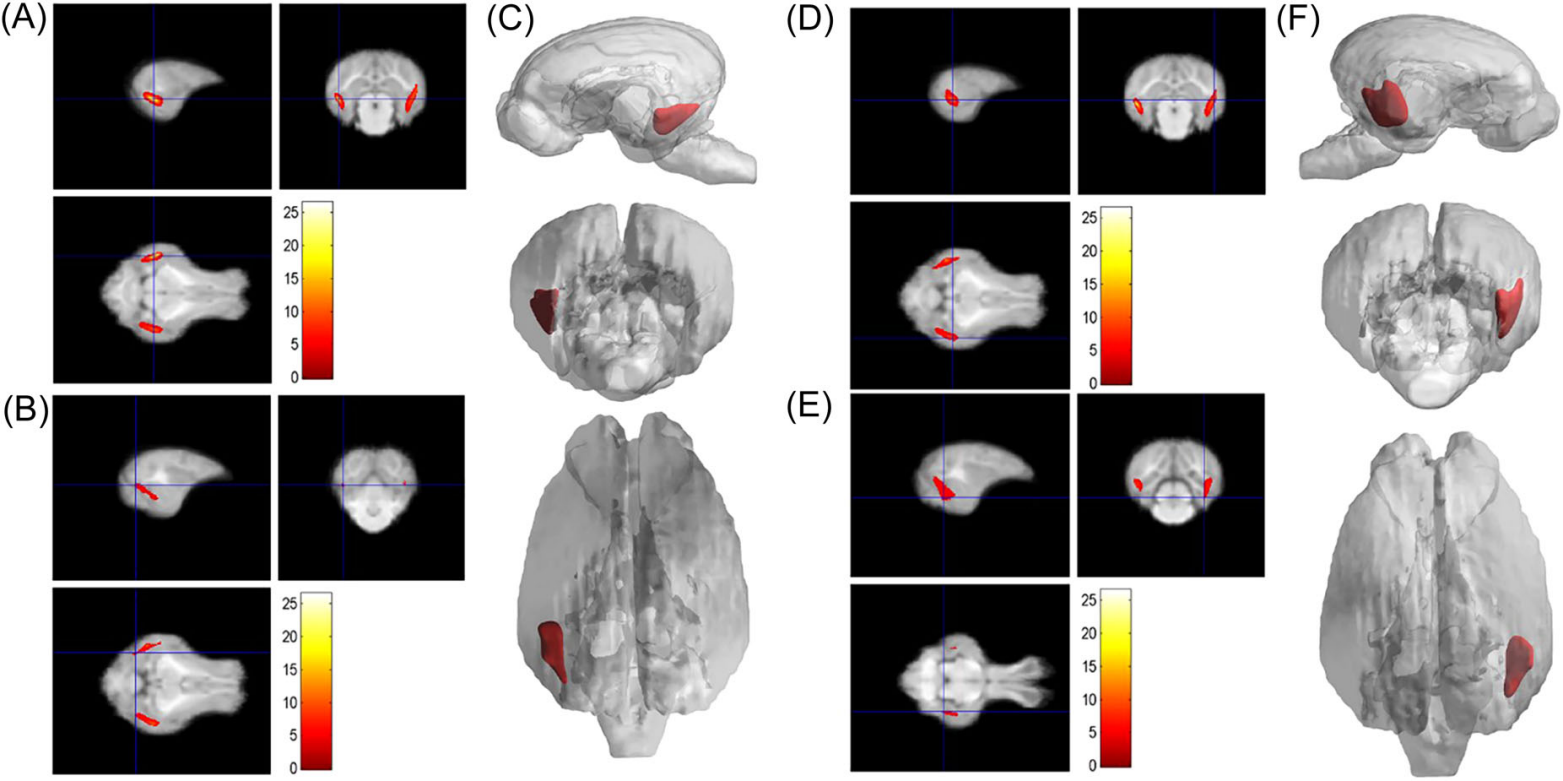
Results of the adult group and the M&O group. (A, B) It was shown that the atrophy in the left temporal lobe and the main peak region (inferior temporal gyrus; *x* = −17, *y* = −4.5, *z* = −0.5; *x* = −14.5, *y* = −12.5, *z* = 2), and the total cluster size was 1342. (C) The 3D reconstruction of the atrophy area of the left temporal lobe. (D, E) The right temporal lobe of cerebellum showed atrophy in the main peak region (inferior temporal gyrus; *x* = 18, *y* = −4, *z* = 1.5; *x* = 14, *y* = −8, *z* = −5.5), and the total cluster size was 2153. (F) The 3D reconstruction of the atrophy area of the right temporal lobe. M&O, middle‐and‐old age pigs. [Color figure can be viewed at wileyonlinelibrary.com]

**Figure 6:**
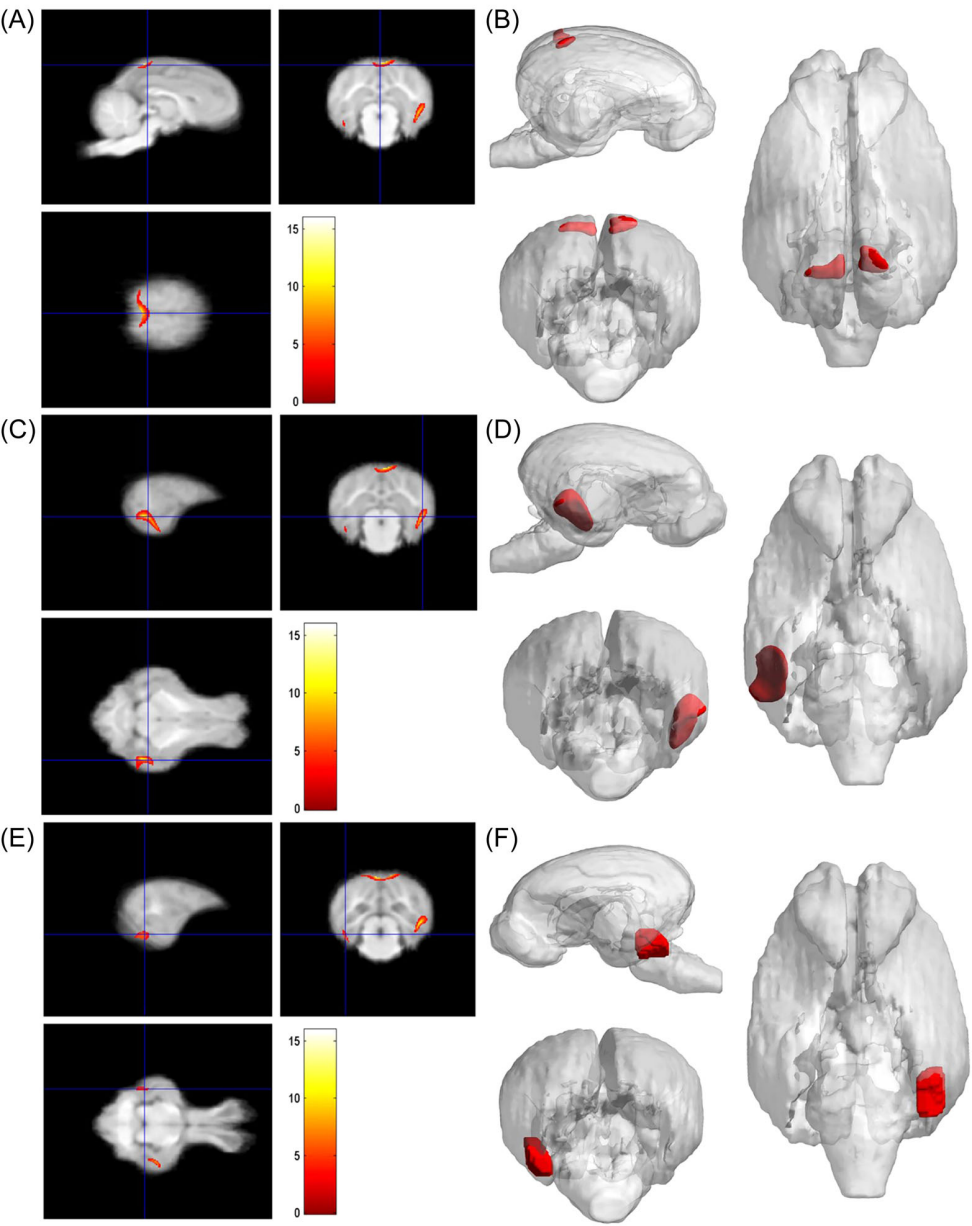
Results of the volume of the adult group and the M&O group. (A) The M&O group showed atrophy on the left and right sides of the brain in the main peak regions (precuneus; *x* = −1, *y* = −6, *z* = 19.5; *x* = −9, *y* = −7.5, *z* = 19.5), and the total cluster size was 701. (B) The 3D reconstruction of left and right precuneus atrophy. (C) The main peak region of the right temporal lobe was the inferior temporal gyrus (*x* = 16.5, *y* = −7, *z* = −2), and the total cluster size was 1244. (D) The 3D reconstruction of right temporal lobe atrophy. (E) The main peak region of the left temporal lobe was the inferior temporal gyrus (*x* = −15.5, *y* = −7, *z* = −8), and the total cluster size was 247. (F) The 3D reconstruction of the atrophy area of the left temporal lobe. M&O, middle‐and‐old age pigs. [Color figure can be viewed at wileyonlinelibrary.com]

As shown in Table 2, compared with the young group, the pigs in the adult group, the main peak region of brain atrophy on the right side was the lingual gyrus (*x* = 16.5, *y* = −3, *z* = −0.5), with a total cluster size of 3953, the main peak region of brain atrophy on the left side was the lingual gyrus (*x* = −14, *y* = −7, *z* = −2.5), with a total cluster size of 3953. In addition, the right precuneus and superior frontal gyrus were also atrophied to a lesser extent (Figure 4). Compared with the young group, in the M&O group, atrophy was observed in the left temporal lobe and the main PEAK region was the inferior temporal gyrus (*x* = −17, *y* = −4.5, *z* = −0.5), the total cluster size was 1342, the main peak region in the right temporal lobe of the cerebellum was the inferior temporal gyrus (*x* = 18, *y* = −4, *z* = 1.5), the total cluster size was 2153 (Table 3 and Figure 5). Compared with the adult group, the main peak region of the left and right cerebral atrophy in the M&O group was the precuneus (*x* = −1, *y* = −6, *z* = 19.5), and the total cluster size was 701. The main peak region of the right temporal lobe was the inferior temporal gyrus (*x* = 16.5, *y* = −7, *z* = −2), and the total cluster size was 1244. The main peak region of the left temporal lobe was the inferior temporal gyrus (*x* = −15.5, *y* = −7, *z* = −8), and the total cluster size was 247 (Table 4 and Figure 6).

**Table 2:** Comparison of young group and adult group

Cluster size	Peak region	MNI coordinate (mm)			*T*	*p* value
		*x*	*y*	*z*		
3953	Right occipital glossal gyrus	16.5	−3	−0.5	20.35	0.0012
2281	Left occipital glossal gyrus	−14	−7	−2.5	7.06	0.0097
814	Right precuneus	0.5	−13	5.5	4.39	0.0238
333	Right superior frontal gyrus	0.5	21	12.5	3.49	0.035

Abbreviation: MNI, Montreal Neurological Institute.

**Table 3:** Comparison of young group and M&O group

Cluster size	Peak region	MNI coordinate (mm)			*T*	*p* value
		x	y	z		
1342	Left temporal lobe inferior temporal gyrus	−17	−4.5	−0.5	26.53	0.0007
	Left temporal lobe inferior temporal gyrus	−14.5	−12.5	2	4.86	0.0197
2153	Right temporal lobe inferior temporal gyrus	18	−4	1.5	10.96	0.0041
	Right temporal lobe inferior temporal gyrus	14	−8	−5.5	5.15	0.0177

Abbreviation: M&O, middle‐and‐old age pigs; MNI, Montreal Neurological Institute.

**Table 4:** Comparison of adult group and M&O group

Cluster size	Peak region	MNI coordinate (mm)			*T*	*p* value
		*x*	*y*	*z*		
701	Precuneus	−1	−6	19.5	15.95	0.0020
	Precuneus	−9	−7.5	19.5	5.57	0.0153
1244	Right temporal lobe inferior temporal gyrus	16.5	−7	−2	11.07	0.0040
247	Left temporal lobe inferior temporal gyrus	−15.5	−7	−8	7.72	0.0081

Abbreviation: M&O, middle‐and‐old age pigs; MNI, Montreal Neurological Institute.

## 4 DISCUSSION

The brain structure of experimental animals is one of their important biological characteristics and is an important index for the modeling and process observation of brain diseases such as cerebral palsy, hypoxic‐ischemic injury, cerebral infarction, and Alzheimer's disease.^12^, ^13^ A number of studies have determined the age‐dependent structural brain features in experimental animals using MRI.^14^ Also, some studies have used MRI to predict functional outcomes in clinically relevant TBI pig models.^15^

Our findings suggested that the brain atrophy and functional decline in Diannan small‐ear pigs were related to age. Brain imaging T2WI signal intensity results showed that most of the T2WI signals in the adult group and the M&O group showed a progressive decrease, including the left cerebrum, right caudate nucleus, cerebellum, right cortex, the left lateral ventricle, midbrain, pons, left and right putamen, globus pallidus, right and left thalamus, left and right olfactory bulb, and left and the right internal capsule. It is possible that the overall T2WI signal is significantly reduced with age as the extracellular fluid in the brain progressive decreases. MRI results showed that progressive brain atrophy was observed in both the left and right sides of the adult and M&O groups compared with the young group, with the main atrophy areas being the lingual gyrus, precuneus, and superior frontal gyrus, indicating that the degree of brain atrophy in Diannan small‐ear pigs was correlated with age. Combined with the results of brain imaging, T2WI signal intensity, and morphology, it showed that there were significant differences in brain signal and contraction degree in different age groups of Diannan small‐ear pigs, suggesting that age was an important interfering factor in the brain development of the pigs. It also provided a good basis for the observation of comparative encephalopathy or progressive encephalopathy in different age groups. In addition, MRI is also recommended as a good choice for detecting brain development in Diannan small‐ear pigs.^16^

Traditional animal models used in the experiment, such as fruit flies, zebrafish, and rodents, such as rats and mice, are often used to study the relevant single effects of a gene on biological pathways in human diseases, but often fail to represent the complexity of human physiology; gorillas, monkeys, and apes cannot be used in large numbers due to strict ethical issues.^17^, ^18^ In recent years, miniature pigs, a new type of laboratory animal with the advantages of high similarity to humans and few ethical issues, have been widely used in medical teaching, cardiovascular research, drug evaluation, and disease infection model.^19^, ^20^ These include the relatively high similarity between the pigs and human organs, the physiology and disease development, as well as the shareability of porcine and human genome, transcriptome, proteome, and other database tools. With the rapid development of genetic research and biotechnology in recent years, it has become feasible to construct transgenic pigs with specific genes that can be used in human models of infectious diseases, such as viruses, bacteria, parasitic transmission, and various tumor models.^21^, ^22^ Besides, there has been a gradual increase in studies related to brain lesions in experimental miniature pigs. In 2014, Matthew proposed that the major brain growth bursts in minipigs extend from prenatal to postnatal, similar to humans, and that their brain convolution patterns, gray and white matter distrivution are so similar to human infants. Thus, minipigs represent a gyrencephalic species similar to humans that could be used in highly controlled experiments to explore neurological diseases.^23^ Gregory Simchick et al. conducted functional MRI and diffusion tensor imaging of 12 enclosed group miniature pigs by independent component analysis and sparse dictionary learning and found that the six resting‐state parameters of the brain of the miniature pigs were similar to those of the human brain.^24^ All of these studies demonstrate the potential of experimental miniature pigs for brain modeling and transformation.

## 5 CONCLUSION

In summary, this study revealed that there were differences in the brain structures of Diannan small‐ear pigs at different ages, and the degree of brain atrophy increased with age, providing the basic data for the establishment of brain disease‐related animal models using Diannan small‐ear pigs.

## AUTHOR CONTRIBUTIONS

Yi‐Fan Liu and Chang‐Le Fang designed the study and wrote the paper. Yu Pi, Teng‐Fei Ke, Ji‐Xiang Chu, Lin‐Na Tang, and Lan‐Chun Zhang performed the experiments and analyzed the data. Somjit Wanchana and Cheng‐De Liao made substantial contributions to revising the manuscript.

## CONFLICT OF INTEREST

The authors declare no conflict of interest.

## ETHICS STATEMENT

The study and all procedures were approved by the Animal Ethics Committee of Kunming Medical University (approval no. kmmu2021570).

## Data Availability

The data sets used and analyzed during the current study are available from the corresponding authors on reasonable request.
